# Widening access to cardiovascular healthcare: community screening among ethnic minorities in inner-city Britain – the Healthy Hearts Project

**DOI:** 10.1186/1472-6963-7-192

**Published:** 2007-11-23

**Authors:** Jeetesh V Patel, Ashan Gunarathne, Deidre Lane, Hoong S Lim, Inessa Tracey, Nimai C Panja, Gregory YH Lip, Elizabeth A Hughes

**Affiliations:** 1 Sandwell Medical Research Unit, Sandwell General Hospital, Sandwell and West Birmingham Hospitals NHS Trust, UK; 2 University Department of Medicine, Sandwell General Hospital, Sandwell and West Birmingham Hospitals NHS Trust, UK

## Abstract

**Background:**

The burden of cardiovascular disease (CVD) in Britain is concentrated in inner-city areas such as Sandwell, which is home to a diverse multi-ethnic population. Current guidance for CVD risk screening is not established, nor are there specific details for ethnic minorities. Given the disparity in equitable healthcare for these groups, we developed a 'tailored' and systematic approach to CVD risk screening within communities of the Sandwell locality. The key anticipated outcomes were the numbers of participants from various ethnic backgrounds attending the health screening events and the prevalence of known and undiagnosed CVD risk within ethnic groups.

**Methods:**

Data was collected during 10 health screening events (September 2005 and July 2006), which included an assessment of raised blood pressure, overweight, hyperlipidaemia, impaired fasting glucose, smoking habit and the 10 year CVD risk score. Specific features of our approach included (i) community involvement, (ii) a clinician who could deliver immediate attention to adverse findings, and (iii) the use of an interpreter.

**Results:**

A total of 824 people from the Sandwell were included in this study (47% men, mean age 47.7 years) from community groups such as the Gujarati Indian, Punjabi Indian, European Caucasian, Yemeni, Pakistani and Bangladeshi. A total of 470 (57%) individuals were referred to their General Practitioner with a report of an increased CVD score – undetected high blood pressure in 120 (15%), undetected abnormal blood glucose in 70 (8%), undetected raised total cholesterol in 149 (18%), and CVD risk management review in 131 (16%).

**Conclusion:**

Using this systematic and targeted approach, there was a clear demand for this service from people of various ethnic backgrounds, of whom, one in two needed review from primary or secondary healthcare. Further work is required to assess the accuracy and clinical benefits of this community health screening approach.

## Background

Cardiovascular disease (CVD) is the leading cause of death in Britain [1], but compared to the general population, the burden is more prominent amongst the country's ethnic minorities [2]. This excess of CVD mortality relates to the morbid affliction of its determinants amongst migrant communities, most notably the influence of diabetes and hypertension, which frequents South Asian (those people originating from the Indian subcontinent, including Indian, Pakistani, Bangladeshi and Sri Lankan groups) and African-Caribbean groups [3-6]. Strategies for the prevention of CVD focus on health promotion and the early recognition, assessment and reduction of risk factors in asymptomatic individuals [7]. CVD risk screening guidance that is specific for ethnic minorities is not established, and there is insufficient evidence to develop a consensus approach for ethnically specific CVD management and prevention.

### An outline of the problem

National guidelines from the National Service Framework [8], the National Institute for Clinical Excellence [9] and the Quality and Outcomes Framework [10] relating to coronary heart disease (CHD) prioritise risk management and amongst those individuals with existing CHD, or co-morbidities. However, specific guidance on the strategies for the assessment of CVD risk in asymptomatic individuals is not available [11]. Previously, research on CVD risk in a random sample of South Asians in Sandwell [12] revealed that more than 50% of hypertensives and diabetics were newly diagnosed, highlighting a missed opportunity for the primary prevention of CVD in this high risk group. By comparing immigrant communities in Britain to their contemporaries living in their country of origin, studies illustrate the insidious change in CVD risk amongst migrants, which is not evident from absolute measures of serum cholesterol, blood pressure and anthropometry in these groups [12-14]. The implication is that the burden of dyslipidaemia, hypertension and diabetes is likely to be underestimated in ethnic minorities in Britain. Moreover, national data suggests that current healthcare services are flawed with regard to equitable healthcare for ethnic communities in Britain [15], with one specific example being the availability and utility of bilingual services [2].

### A culturally specific approach

The initiative to begin health screens within local communities was first realised as an outcome of ongoing research in a cohort of randomly selected Indian Gujaratis in 2002 [12]. This was followed by an 'ad hoc' two-year programme of 'in kind' CVD risk screens within the broader South Asian communities and the general population of Sandwell. With experiences gleaned from these screens, the following features were deemed essential for the success of any community health screening event: (i) the involvement of the local community to develop and advertise the health screening events, (ii) the presence of an experienced physician with special interest in CVD (to deliver immediate attention to adverse findings), and (iii) the use of an interpreter and multi-lingual staff, who, where possible, were available to assist prospective participants to make informed choices in their involvement in the health screen, and improve communication during consultations.

The key anticipated outcomes were the numbers of participants from various ethnic backgrounds attending the health screens and the prevalence of known and undiagnosed CVD risk within ethnic groups. The evidence collected would then be used to determine whether it was feasible to adopt community health screening events into primary prevention services that operate to reduce CVD risk.

## Methods

### Setting

In the UK, CVD mortality rates in inner city areas such as Sandwell are the highest nationally for England and Wales [1]. Information from the 2001 census reveals that between 20 and 25% of the 300,000 population of Sandwell are from ethnic minority groups (including Bangladeshi (1.21%), Indian (9.14%) and Pakistani (2.95%) populations). Sandwell is amongst the most severely deprived areas in the UK, with the Carstairs Index, Townsend Score, Underprivileged Area Index and Local Deprivation Index all in the upper quintile for the majority of the electoral wards [1]. Compared nationally, health in Sandwell is generally poor, where fruit and vegetable consumption are significantly lower, whilst smoking and obesity rates are markedly higher in the adult population [16].

### Development of Health Screening Events

Given the burden of CVD in the area, and the broad multi-ethnic population, a Community Health Network (CHN), a public health initiative team from Sandwell Primary Healthcare Trust, who respond to healthcare concerns and needs raised by Black and Minority Ethnic (BME) groups, operate in the locality. The CHN team was engaged in this 'Healthy Hearts Project' to ensure the integration of local communities (Bangladeshi, Indian Gujarati, Pakistani, Indian Punjabi and the Yemeni) in the development and advertisement of health screening events. Specifically, CHN leads for each community promoted health screening events within the main ethnic minority groups in Sandwell: Bangladeshi (3 events), Pakistani (1 event), Gujarati (1 event), Indian Punjabi (2 events) and Yemeni (1 event) within community venues. The promotion of the health screening event included posters (translated as appropriate) within community halls, local GP clinics and healthcare centres (through the Primary Care Trust) and the advertisement of the event in the form of circulars distributed by the community (2 weeks before the event by the CHN leader). Subjects were advised to come fasting (no food or drink from 10 pm the previous evening) if they wanted their blood sugar assessed as part of a diabetes risk assessment. It was anticipated that 100 individuals would attend each event. Both the circulars and the posters contained identical information, requesting men and women aged from 25 years to attend health screening events. In addition, there were two events within public spaces (Sandwell Community show) that focussed on the attendance of the general European Caucasian population (advertisement was through similar posters distributed to local GP clinics and healthcare centres, with circulars distributed on the day of the event). Our target population of 1000 people was to include those people who were representative of men and women within the five main ethnic groups in Sandwell (100 in each gender group). Using this targeted and systematic approach, these series of ten community health screening events, between September 2005 and July 2006, called the 'Healthy Hearts Project', were conducted to collect information on the performance of these health screens within ethnic communities.

### The 'Healthy Hearts' Team

The staff of the Healthy Hearts team were composed of health professionals working within the Sandwell and West Birmingham Hospitals NHS Trust, specifically Sandwell Medical Research Unit and the Haemostasis, Thrombosis and Vascular Biology Unit. The staff of the healthy hearts team varied in accordance to the specific needs of the community group targeted (i.e an all-female team was a requisite for two events). The Healthy Hearts team included a hospital-based interpreter or an appropriate CHN leader to facilitate the informed consent process. The team consisted of physicians, nurses, and researchers, who were experienced in the assessment of CVD risk.

### Procedure

The health screening events were started between 8 am to 9 am, and lasted for five hours. People attended the health screening event in a "fasting" state. Firstly, written informed consent was obtained, followed by demographic information including their fasting status, ethnicity, general practitioner details (where the subject had consented for this information to be included) and a medical history questionnaire relating to cardiovascular health and diabetes.

Following consent, participants were invited to be assessed for the measurement of body weight (Seca floor scales) and height (Leicester height measure, Seca Ltd, Birmingham UK), where the subject was measured without shoes in light clothing, and advised to stand in a posture to accommodate their heads to be in the vertex plane for height assessment. Body mass index was calculated as weight(kg)/height(meters)

squared. Capillary blood cholesterol and glucose were measured using the Roche Accutrend GC monitor (Roche Diagnostics, Welwyn Garden City, UK), using methodology approved by the Royal Pharmaceutical Society of Great Britain (2003). The medical history questionnaire was completed by the nurse or researcher (with interpreting services used as appropriate), and the blood pressure was measured after the patient was seated for more than five minutes using the OMRON 705CP (Omron Healthcare Europe, Mannheim, Germany), a semi automated blood pressure monitor used with appropriate cuff sizes. The blood pressure was measured in triplicate in accordance with the British Hypertension Society guidelines [17], where the mean of the last two measurements was used as the final reading. Hypertension was defined by either self-reported history (participants knowledge of a diagnosis of hypertension by a doctor or a health professional), or the use of antihypertensive medication or a systolic blood pressure > 140 mmHg, or a diastolic blood pressure > 90 mmHg. Current and previous smoking habit (more than 100 cigarettes smoked in total) was assessed amongst both men and women. New hyperlipidaemia was defined as a total cholesterol > 5 mmol/l and the absence of any history of lipid lowering medication. Diabetes was defined as a self-reported history (participants knowledge of a diagnosis of diabetes by a doctor or a health professional) and newly detected glucose intolerance was defined as a fasting capillary blood sugar > 6 mmol, and the absence diabetes or use hypoglycaemic medication/insulin.

Using the coronary risk calculator of the European Society of Cardiology (ESC) the absolute risk (%) of developing non fatal coronary heart disease or coronary death over the next 10 years was estimated. This algorithm was derived from the European risk charts derived from SCORE estimate risk of cardiovascular death, based on the risk factors: age, gender, smoking habit, blood pressure and blood cholesterol [18].

The information was recorded on the proforma, and transcribed onto a results sheet for the participant by the physician. The results were explained to the participant by the physician (using the interpreter as appropriate) who gave general lifestyle advice for those with blood pressure, anthropometrical or biochemical measures that indicated a very low or borderline CVD risk based on Joint British Society (JBS 2) [7]. For all those asymptomatic participants with an increased CVD risk score (more than 15%), or BMI > 27 kg/m^2^, or systolic blood pressure > 140 mmHg or diastolic blood pressure > 85 mmHg (in the absence of established atherosclerotic disease, diabetes, and chronic renal failure), or total cholesterol > 5 mmol/l or diabetes risk (fasting/non-fasting blood glucose > 5 mmol/l), repeat clinical investigations were organised and the physician developed a management plan that involved both primary (with culturally sensitive options for education, lifestyle related risk management and prevention provided by the CHN leader) and secondary care as appropriate. The criteria for management review was based on JBS2 guidelines, where those individuals with a total CVD risk score more than 20% over 10 years were defined as high risk, and were highlighted for professional lifestyle intervention. In addition, smoking cessation was advised to all smoking participants, where the CHN lead was able to advise culturally targeted options. The consent process and proforma were approved by the Sandwell Local Research Ethics Committee.

### Analytical methods

All data (where the participant had agreed consent) were entered for analysis into a statistical database, SPSS v14 (SPSS Inc., Chicago, IL). Continuous data are presented as mean (standard deviation) and comparisons of means were analysed by t-test between two independent groups (eg gender) or ANOVA for multiple groups (ethnic group), using Tukey for post Hoc analysis. The distribution of all participants within an age stratification (25–34, 35–44, 45–54 and 65+ years) was used to determine the age-adjustment needed of all categorical variables within ethnic groups. An estimate of the 10-year Cardiovascular Risk Score was made using information gleaned from the screening process. An estimated HDL cholesterol level of 1 mmol/l was used in all the subjects in calculating the 10 year cardiovascular risk.

## Results

### Participation within the health screens

A total of 824 (47% male) individuals who attended the health screens consented for their information to be analysed. The information was collected from 10 community health screen events, where community groups such as the Gujarati Indian, European Caucasian, Punjabi Indian, Yemeni, Pakistani and Bangladeshi were targeted. Of these targeted community groups, the attendance at Gujarati Indian, Punjabi Indian and Yemeni health screening events was good, with approximately 80 people (not all people consented for their data to be analysed) attending. Attendance amongst Bangladeshi and Pakistani groups was poorer, where less than 50% of our anticipated attendance was met. The health screening events were not exclusive for any particular ethnic group and an additional category of 'Other Indian subcontinent' (mostly Sri Lankan, South Indian) are included, which are merged with the Pakistani cohort (37 individuals) in this analysis. The largest group who attended the Health screens were the European Caucasian population (276 out of an anticipated 200 people). A total of ten individuals were of Caribbean origin, and are not included in the analytical analysis due to the size of the group.

### Demography and management review

The age range amongst these individuals was 25 to 90 years. The mean (SD) age of the total population was 47.7 (15.2) years, with no significant variation by gender: 48.7 (15.2) years in men, 46.9 (15.1) years in women, (*P *= 0.09). Across ethnic groups, the mean age differed (*P *= 0.006). The 'Other Indian subcontinent' group (mean 42.4 years, 95%CI 39.1–45.6) was the youngest compared to all other ethnic groups, in whom age was comparable (i.e there were no differences in age between Caucasian, Gujarati, Bangladeshi, Yemeni and Punjabi). A total of 185 individuals attended the health screening events in a fasted state, and fasting rates were comparable between ethnic groups (10–20%), save the Bangladeshi group in whom 40% attended in a fasting state. With regards to smoking, all those with a positive response were exclusively current smokers, where age adjusted rates amongst men (13.4%(95%CI 10.0–16.8)) were almost double those reported in women (7.7%, (5.2–10.4)). In addition, smoking habit was generally absent amongst Gujarati, Punjabi, Bangladeshi women and Yemeni women, and highest amongst Bangladeshi men. A total of 470 (57%) individuals were referred by the Healthy Hearts physician to their general practitioner (there were no cases where a subject did not have a general practitioner) with a report of an increased CVD risk, (undetected high blood pressure in 120 (15%), undetected abnormal blood glucose in 70 (8%), undetected raised total cholesterol in 149 (18%), and CVD risk management review in 131 (16%)).

### Cardiovascular risk profile in communities (Tables 1 and 2)

**Table 1:** Age adjusted prevalence of cardiovascular risk factors and 10-year coronary heart disease risk score* in men by ethnic group

Age-adjusted prevalence of cardiovascular risk factors in men	Gujarati Indian (n = 43)	Punjabi Indian (n = 87)	European Caucasian (n = 119)	Bangladeshi (n = 66)	Yemeni (n = 32)	Other Indian Subcontinent (n = 37)
Known hypertensive	30.2 (16.5–44.0)	27.6 (18.2–37.0)	16.1 (7.2–25.0)	16.1 (7.2–25.0)	26.2 (12.6–43.8)	39.2 (23.4 – 54.9)
Newly diagnosed hypertensive	32.2 (18.2–46.2)	22.0 (13.3–30.8)	29.0 (20.8–37.2)	7.0 (0.8–13.2)	19.3 (5.6–33.0)	24.3 (10.5–38.1)
Known diabetic	11.6 (2.0–21.2)	15.6 (6.7–24.5)	2.6 (0–5.5)	26.3 (15.5–37.0)	9.4 (0–19.6)	0
Newly diagnosed glucose intolerance (fasting blood glucose > 6 mmol/l)	7.6 (0–15.6)	0	0.7 (0 – 2.1)	8.8 (1.9–15.8)	3.4 (0–9.8)	6.6 (0–15.7)
Known hyperlipidaemia or current cholesterol lowering therapy	7.3 (0–15.3)	5.7 (0.9–10.6)	1.7 (0 – 4.0)	1.3 (0–4.1)	11.7 (0.5 – 22.8)	3.2 (0–9.1)
Newly diagnosed raised total cholesterol (>5 mmol/l)	21.1 (8.7–33.7)	11.6 (4.9–18.4)	24.0 (16.7 – 31.7)	13.2 (5.0–21.4)	21.3 (7.1 – 35.5)	25.9 (11.4–40.4)
Body mass index > 27 kg/m^2^	43.1 (28.0–58.3)	40.0 (29.7–50.3)	48.4 (39.4–57.4)	48.2 (36.1–60.2)	45.7 (28.5–63.0)	56.2 (39.7–72.6)
Current smoking habit	2.3 (0–6.8)	0	14.7 (21.2)	28.7 (17.8–39.6)	25.0 (9.8–10.3)	11.4 (0.5–22.2)
CHD risk score	10.7 (9.3)	7.6 (6.1)	8.5 (7.1)	9.0 (8.3)	8.5 (6.7)	9.1 (9.0)

Data are percent (95%CI) except *CHD risk score which is mean (SD), calculated with a HDL cholesterol of 1 mmol/l for all subjects. Other Indian subcontinent includes Pakistani (17 men), Sri Lankan and South Indian.

**Table 2:** Age adjusted prevalence of cardiovascular risk factors and 10 yr coronary heart disease risk score* in women by ethnic group

Age adjusted prevalence of Cardiovascular risk factors in women	Gujarati Indian (n = 36)	Punjabi Indian (n = 76)	European Caucasian (n = 157)	Bangladeshi (n = 94)	Yemeni (n = 36)	Other Indian Subcontinent (n = 31)
Known hypertension	26.9 (12.4–41.3)	15.2 (7.1–23.2)	12.5 (7.2–17.7)	20.0 (11.9–28.1)	29.6 (14.7–44.5)	5.4 (0–26.7)
Newly diagnosed hypertension	15.0 (3.3–26.7)	11.3 (4.2–18.5)	21.3 (6.7–0.5)	13.6 (6.7–20.5)	10.5 (0.5–20.5)	14.1 (0–2.3)
Known diabetes	6.0 (0–13.7)	6.4 (0.1–12.8)	1.7 (0–3.8)	21.3 (13.0–29.6)	13.0 (2.0–23.9)	7.8 (0–10.5)
New glucose intolerance (fasting blood glucose > 6 mmol/l)	16.1 (4.1–28.1)	0	0	5.5 (0.9–10.1)	0	2.2 (0–3.1)
Known hyperlipidaemia or cholesterol lowering therapy	7.8 (0–16.8)	7.1 (1.3–12.8)	2.1 (0–4.4)	8.2 (2.7–13.8)	8.8 (0–18.1)	8.7 (0–47.8)
Newly diagnosed raised total cholesterol (>5 mmol/l)	32.2 (16.6–48.0)	14.0 (6.2–21.8)	36.8 (29.1–44.5)	17.2 (9.6–24.8)	11.2 (0.9–21.4)	17.6 (0–70.5)
Body mass index > 27 kg/m^2^	37.6 (21.4–53.9)	50.1 (38.8–61.3)	43.7 (35.8–51.5)	47.2 (37.1–57.3)	49.5 (33.1–65.8)	41.0 (0–109)
Current smoking habit	0	3.1 (0–7.0)	18.8 (12.6–25.0)	0.8 (0–2.6)	0	0
CHD risk score	6.89 (8.35)	6.60 (5.77)	5.56 (5.27)	6.60 (7.00)	5.60 (8.29)	4.39 (5.15)

Data are percent (95%CI) except *CHD risk score which is mean (SD), calculated with a HDL cholesterol of 1 mmol/l for all subjects. Other Indian Subcontinent includes Pakistani (20 women) and South Indian.

Within combined communities the overall prevalence of hypertension was 39.2% (95%CI: 35.9–42.5), half of whom (19.6% (16.9–22.3)) were newly detected, with no significant differences in prevalence between different ethnic groups. Known hyperlipidaemia or active lipid lowering therapy was reported by 4.2% (2.8–5.6) of the total population, where 22.6% (19.7–25.5) of all people screened had newly detected raised total cholesterol (>5 mmol/l) with no significant ethnic variation. The prevalence of known diabetes and impaired fasting glucose were at least 3 times more common in Punjabi, Gujarati, Bangladeshi and Yemeni groups compared to the European Caucasian population. A total of 555 (67%) individuals who attended the health screens were aged 55 years or younger. Among these younger participants, 123 (22%) had newly diagnosed raised cholesterol, 89 (16%) had newly diagnosed high blood pressure (16%) and 58 (11%) had newly diagnosed glucose intolerance.

## Discussion

Strategies for the prevention of CVD aim to reduce the prevalence of associated risk factors, particularly amongst asymptomatic people at high risk of developing or worsening CVD. Screening for, and treatment of, dyslipidaemia and hypertension appear to return the largest clinical benefit compared to the detection of other less tangible CVD risk factors (albeit to the health professional), such as smoking habit, diet and anthropometry [19] (specifically, smoking habit and diet assessment are susceptible to reporting bias, and appropriate approaches for body weight and shape cut-offs fuel increasing debate in Asian countries). However, direct data from CVD risk screening are not available amongst South Asian or Yemeni groups, and data from the general population would suggest that such screening approaches have limited benefit with respect to improved CVD management [20]. A recent review investigated the accuracy of CVD risk scoring and reported that the risk prediction scores generally overestimated observed risk in low risk populations and that there was no strong evidence that CVD risk assessment performed by the clinician improved health outcomes [11]. However, in high risk populations such as patients with diabetes [21], or raised socioeconomic deprivation [22], CVD risk scoring actually underestimated risk. Hence, within a multi-ethnic inner – city area such as Sandwell, many high risk individuals may not reach the treatment threshold. A calibration of the CVD risk score within areas with high rates of CHD is needed [21].

With specific reference to the JBS2 recommendations in 2005, we would propose that these health screens are not limited by approaches that focus on the elderly and should target community groups using multi-lingual teams.

The uptake of interpreter services was not assessed in this study. However, amongst the Gujarati, Bangladeshi, Pakistani, Punjabi and Yemeni health screening events, interpreters and multi-lingual staff were required for the informed choice process and physician consultation in more than half of the people that attended. Moreover, we did not collect information on second- and third-generation migrants born in the UK, who are less likely to exhibit the early reported CVD-protective traits, such as low serum cholesterol and body mass index of the first generation (born overseas) [23].

Outcomes associated with service delivery arising from this work are to examine the logistics of establishing screening programmes to detect hypertension, diabetes and other CVD risk factors, which are based within the community utilising community and religious facilities where the population naturally congregate. By working with community leaders, the Healthy Hearts project has developed an effective method of organising health screening events, not only identifying individuals at risk, but raising awareness of diabetes, high blood pressure, heart disease and stroke by health promotion. Further work is needed to assess the integration of 'at risk' individuals into existing channels of healthcare once cardiovascular problems are highlighted in the community setting. In the present project, the CHN leaders were present to consolidate appropriate lifestyle management advice given by the physician, though their prospective attendance at such programmes was not recorded.

Of note, while rates of diabetes and impaired fasting glucose were more common amongst the South Asian and Yemeni groups, the prevalence of undetected hypertension and hyperlipidaemia were comparable across all groups, which suggests equity in healthcare provision, albeit for this high risk region. The Health Survey for England, which includes information gleaned from a representative sample survey in 2004 [24] reflects the known rates of diabetes amongst South Asian groups reported here, but levels of hypertension and hyperlipidaemia were markedly higher amongst our Bangladeshi group. Rates of smoking were also higher amongst the Bangladeshi male group, who are the most likely group in Britain to smoke [1]. The attendance of Bangladeshi and Pakistani groups at health screening events was considerably less compared to other ethnic groups targeted. The reasons for this are not clear, particularly as we included gender specific health screening events to be sensitive to possible cultural mannerisms. However, this result highlights the importance of developing subsequent programs for better community outreach that will target hard-to-reach subgroups"

The health screening events were started in the morning at a weekend, which may not have been appropriate for many Bangladeshi and Pakistani men, who characteristically work night shifts in the taxi and restaurant businesses. Of interest, rates of fasting attendance were higher amongst the Bangladeshi group, and this may be a source of bias in their attendance and the interpretation of glucose intolerance. In previous research by our group amongst a representative sample (using random sampling of the electoral register) of male and female Gujaratis from the same community presented here [12], we reported higher rates of known diabetes, current smoking habit unknown dyslipidaemia and similar rates of hypertension (though rates of known hypertension were much higher in the present study).

Limitations in our approach include the use of the Framingham risk equation, where recalibration is needed for this region of high deprivation and ethnic diversity. The QRISK equation [25], which identifies and includes deprivation in the estimation of CVD risk and ETHRISK [26], a modified Framingham CVD risk equation for Black and Minority groups in Britain were not available at the time of the project, but would have represented a more appropriate option for risk estimation. Moreover, the use of 1 mmol/l as a universal measure of HDL cholesterol amongst both men and women is likely to contribute to an overestimation of risk amongst in this project. However, given our approach to provide health screening participants with immediate advice in relation to CVD risk, it was necessary to estimate a CVD risk score, even in the absence of a HDL cholesterol measurement (which was not available as a validated capillary sample approach at the time of the project). The first line of management of diabetes, raised cholesterol and hypertension is that of diet and lifestyle changes. From our research in the Gujarat [12], it was evident that the diet within the various communities had changed from traditional practices to one of higher fat intake and increasing reliance on processed foods. Of note, dietary advice that was culturally sensitive was not available at the time of this project for many of the individuals that attended health screening events.

## Conclusion

Within the communities assessed, the present data show that there is a demand and need for the assessment of diabetes, hypertension, and CVD risk in adults, even among younger people, from the age of 25 years. Using this systematic and targeted approach, an average of eighty people from various ethnic backgrounds attended the one-day screening events, of whom half needed review from primary or secondary healthcare. Importantly, the approach engaged the local communities in health promotion and allowed immediate medical attention to reduce CVD risk. The implication of these findings is that there is a role for this targeted approach to CVD risk screening in high risk areas such as Sandwell to widen the access of the community to existing primary and secondary CVD healthcare services and promote healthy living.

## Competing interests

No competing interests for HS Lim, D Lane, I Tracey, N Panja, A Gunarathne and GYH Lip. Both JV Patel and EA Hughes have received funds from pharmaceutical companies (Astra Zeneca, Sanofi Aventis, Solvay Healthcare) to conduct this work.

## Authors' contributions

JVP, HSL, DL, IT, NP, AG, EAH conducted the health screens, JVP, AG and DL drafted the manuscript. EAH and GYHLIP participated in the design of the study and assisted the conception, and its coordination. EAH and JVP contributed to the funding of this research.

## Pre-publication history

The pre-publication history for this paper can be accessed here:


